# Shoulder complaints in wheelchair athletes: A systematic review

**DOI:** 10.1371/journal.pone.0188410

**Published:** 2017-11-21

**Authors:** Omar W. Heyward, Riemer J. K. Vegter, Sonja de Groot, Lucas H. V. van der Woude

**Affiliations:** 1 University of Groningen, University Medical Center Groningen, Center for Human Movement Sciences, Groningen, The Netherlands; 2 Amsterdam Rehabilitation Research Centre, Reade, Amsterdam, The Netherlands; 3 University of Groningen, University Medical Center, Center for Rehabilitation, Groningen, The Netherlands; University of Illinois at Urbana-Champaign, UNITED STATES

## Abstract

**Background:**

In recent years the popularity of disabled sports and competition among disabled athletes has grown considerably. With this rise in exposure of, and participation in wheelchair sports comes an increase in related stressors, including musculoskeletal load. External mechanical loading may increase the risk of shoulder complaints. The objective of this literature review was to 1) identify and describe the prevalence and/or incidence of shoulder complaints in wheelchair athletes in the literature, to 2) examine the factors and underlying mechanisms that could be potentially involved, and 3) provide some insights into the development of preventative measures.

**Methods:**

A literature search was conducted using PubMed, Scopus and Embase databases, to identify relevant published articles. All articles in the English language that contained any type of shoulder complaint in relation with a wheelchair sports player, at any level of status (recreational to elite), of any sport, were included. Articles were excluded if they did not include any statistical analysis. Articles that included studies with wheelchair athletes in combination with athletes of other disability sports were excluded in order to be able to differentiate between the two. Narrative, exploratory and case studies were also excluded. Two reviewers independently assessed articles for inclusion. Thirteen articles matched the selection criteria. These were judged on their quality by use of an adapted version of the Webster checklist.

**Results:**

Of the included studies the overall quality was low. A relatively high prevalence of complaints was found, ranging from 16% to 76%. Pain was found to be a common complaint in wheelchair athletes. Based on the current literature the cause of shoulder problems is difficult to identify and is likely multifactorial, nevertheless characteristics of the user (i.e. increased years of disability, age and BMI) were shown to increase risk. Preventative measures were indistinct. There may be a role for balanced strength training regimens to decrease risk.

**Conclusion:**

Shoulder complaints in wheelchair athletes are a common problem that must be addressed further. Future studies on shoulder overuse injuries of wheelchair athletes should be directed towards biomechanical modeling to develop knowledge of load and its effects.

## Introduction

In recent years the popularity of disabled sports and competition among disabled athletes has grown considerably. The summer Paralympic Games has had 42 more countries participating between the years 2000 to 2012 [1]. From 2012 to 2015 alone the International Paralympic Committee (IPC) has seen an increase in the number of licensed athletes for numerous sports including an increase of 1156 for athletics, 112 for ice sledge hockey, 476 for powerlifting, 116 for shooting, 533 for swimming, and 156 in wheelchair dance sport [1,2]. Many disability sports are played in wheelchairs with wheelchair basketball, racing, rugby and tennis being commonly played sports. With this rise in exposure of, and participation in wheelchair sports comes an increase in related stressors and complaints; this impacts particularly the shoulder complex [3]. A recent descriptive epidemiology study found parallel evidence that the shoulder is the most common site of complaints among wheelchair athletes [4].

The shoulder complex is a particularly sophisticated and fragile system. In the context of disability, and especially in manual wheelchair users, the upper body and shoulder complex are utilized in almost all tasks of both sports and activities of daily living. Therefore appropriate functioning of the shoulder complex holds the utmost importance to upholding quality of life (QoL) for many individuals. The shoulder complex is a multifaceted system that relies upon the smooth coordination of the scapula, humerus, clavicle and thorax in combination with the associated scapula-humero-thoracic musculature for optimal functioning [5]. The shoulder complex affords large amounts of mobility for the hands due to the functional nature of the structures involved. There is a fine interplay between mobility and stability; the shoulder complex must be mobile enough to allow a full range of motion but simultaneously be stable enough to maintain sufficient integrity and to organize external forces[6].

Load may be an important characteristic in association to complaints. The assumption from musculoskeletal and occupational biomechanical studies is that excessive load may have deleterious effects. An increase in load on the shoulder complex may be considered to be detrimental to its integrity [6–11]. This could be analyzed via the model of Hoozemans [7] who described ‘external load’ to be expressed by three factors: intensity (amplitude and direction of force), frequency and duration. Each of these factors has an optimal value. If any of the factors sway too far from their optimal value, or the combination of the submaximal values from the three factors is increased, the risk of complaints is by default also increased [7,12].

The ‘external load’ upon the shoulder complex is imposed by the characteristics of the task and user. In the case of wheelchair athletes, the characteristics of the task include wheelchair propulsion, activities of daily living (ADLs) and the demands of the specific sport. Steady-state wheelchair propulsion, even during low intensities, has been shown via means of biomechanical modeling studies to generate large loads in the scapula-humero-thoracic musculature [9,11]. Wheelchair ADL tasks have also been shown to generate high loads in the shoulder [9,13]. Characteristics of the specific sport (e.g. ball handling, overhead activities etc.) could also play a role in increasing load on the shoulder complex [14,15]. For instance, the racket hand in wheelchair tennis needs to deliver higher peak forces due to the reduced hand-racket coupling to the handrim [16].

The user specific factors that could play an important role in the expression of shoulder complaints are time since disability, age, gender, body mass index (BMI) and training status [14,17–20]. The type of disability an athlete has may predispose them to a higher risk of complaints than other populations. Much of the research in non-athletic manual wheelchair users studies persons with a spinal cord injury (SCI). People with an SCI, especially those with a longer time since injury, are at increased risk for musculoskeletal and other complaints compared to persons with a shorter time since injury [17]. Increased age and weight have also been shown to be factors that influence shoulder complaints in manual wheelchair users [18,19]. Additionally there may be a gender difference in which more females are affected by shoulder complaints when compared with males but this association is not clear [20]. Furthermore, with respect to training status, trained wheelchair athletes may experience less pain than their nonathletic counterparts. A study of Fullerton et al. [14] described that there may be a protective mechanism of athletic activity on the shoulder complex. In this study it was illustrated that individuals who participate in wheelchair sports are able to live more years without shoulder pain than non-athletes.

Shoulder complaints are a common predicament in able-bodied athletic populations and manual wheelchair users alike. Complaints have been well documented in able-bodied athletic populations in many sports including, but not limited to, baseball [21], swimming [22], water polo [23] and volleyball [24], and have been attributed to a multitude of factors, including impingement syndrome. There has been recent debate as to whether impingement syndrome is the most appropriate diagnostic label [25]. Subacromial pain may better encapsulate the syndrome. As much of the following research was published prior to this scientific debate both terms have been used in this systematic review. Subacromial pain syndrome is one of the most common pathologies of the shoulder in sports medicine and may be the result of rotator cuff pathology, increased shoulder mobility, scapular dyskinesis, muscular imbalances, biceps pathology, labrum tear, glenohumeral internal rotation deficit or repetitive movements [22–24,26–31].

It can be seen that the shoulder is a common site of injury and our current understanding of the cause of shoulder complaints in wheelchair athletes is limited. A comprehensive current literature review is due to synthesize and streamline current knowledge of shoulder complaints in wheelchair athletes. Research should be placed within conceptual frameworks in order to foster organization and develop understanding. Van Mechelen et al.[32] proposed a four-pronged approach to dealing with sports injuries that has been widely utilized in sports medicine literature. Hoozemans et al. [7] has described a conceptual framework (based off the work of Westgaard & Winkel [33] and van Dijk et al. [34]) in which external exposure is related to internal exposure which eventuates to long term effects. The model of Hoozemans et al. [7] may be one in which the problem of shoulder complaints can be placed within in order to break down the problem into manageable chunks in terms of understanding the problem. In accordance with the Van Mechelen et al. [32] model the aim of the current review is to 1) identify and describe the prevalence and/or incidence of shoulder complaints in wheelchair athletes in the literature, to 2) examine the factors and underlying mechanisms that could be potentially involved, and 3) provide some insights into the development of preventative measures.

## Methods

Except for pre-registration, this review followed the Preferred Reporting Items for Systematic Reviews and Meta-Analysis (PRISMA) guidelines[35] (S2 Table).

### Eligibility criteria and study selection

Articles for the review were extracted in 2 levels of study screening. At level 1 screening, titles and abstracts were reviewed by two independent reviewers; decisions were based on the following criteria:

Inclusion criteria: English language articles that contained any type of shoulder complaint in relation with a wheelchair sports player, at any level of status (recreational to elite), of any sport.
Exclusion criteria: Articles that included studies with wheelchair athletes in combination with athletes of other disability sports were excluded in order to be able to differentiate between the two. Narrative, exploratory and case studies were also excluded. Articles were excluded if they did not include any statistical analysis or were written in a language other than English.

A consensus meeting took place if there was a disagreement between both reviewers and they decided together whether to include or exclude the article for the full-text phase. When no consensus could be reached a third author made a binding judgment.

At level 2, one reviewer performed the selection based on full texts. The full text was obtained if the abstract met the in- and exclusion criteria or when there was not enough information available in the abstract to exclude it. When shown in the full-text that an article did not comply with the previous criteria it was excluded. Of the articles included in the review all reference lists were scanned for other relevant articles that may have been missed in the search. Data extraction and methodological quality evaluation was performed on the included articles.

### Search strategy

A systematic search strategy was conducted to identify relevant published articles on the topic of shoulder complaints in wheelchair athletes. PubMed, Scopus and Embase databases were searched for relevant articles published between January 1^st^ 1990 and March 3^rd^ 2017. For the purpose of this study a ‘wheelchair athlete’ was defined as a person with a disability who engaged in regular competitions in a wheelchair sport, the wheelchair did not have to be the athletes only mode of transport. The level of the athlete could range from recreational to elite status. The PubMed search strategy is outlined below, the same strategy was adapted for the other databases.

1. “Shoulder Joint” [MeSH] OR “Rotator Cuff” [MeSH] OR “Acromioclavicular Joint” [MeSH]
2. Shoulder [tw] OR Upper Extremity [tw]
3. (#1 OR #2)
4. “Shoulder Pain” [MeSH] OR “Pain” [MeSH] OR “Syndrome” [MeSH] OR “Wounds and Injuries” [MeSH] OR “Pathology” [MeSH OR “Athletic Injuries” [MeSH] OR “Sport Medicine” [MeSH] OR “Shoulder Impingement Syndrome” [MeSH]
5. Pain* [tw] OR Complaint* [tw] OR discomfort* [tw] OR Injur* [tw]
6. (#4 OR #5)
7. “Wheelchairs” [MeSH] OR “Sports for Persons with Disabilities” [MeSH] OR “Disabled Persons” [MeSH] OR wheelchair [tw]
8. “Sport” [MeSH] OR “Athletes” [Mesh] OR Sport* [tw] OR Athlet* [tw]
9. (#7 AND #8)
10. (#3 AND #6 AND #9)

### Data extraction and quality assessment

The included studies were analyzed independently to extract the following information: participant description (including gender, age, years of athletic activity, sport and level of competition), where the sample was drawn from, study design, whether questionnaires or interviews were utilized, objective measures, clinical evaluation, type of complaint, proposed mechanism and possible preventative measures. This review provides a descriptive summary of the included empirical studies. We refrained from statistically combining results from the different cohorts due to the differences in design.

Assessment of quality was performed independently with an adapted version of a checklist (S1 Table) developed by Webster et al.[23] who also performed a similar study. This checklist was chosen because there is no standardized checklist available for this type of study. The checklist was adapted by one author in order generalize the checklist for the purpose of this review. For each question a score of 1 was given for an ‘adequate’ or ‘yes’ response, a score of 0.5 was given for a ‘partial’ or ‘limited’ response; and a score of 0 was awarded for a ‘no’, ‘not stated’ or ‘inadequate’ response. A maximum score of 8 was possible. There were no minimum criteria set due to the limited amount of papers that were included in the study.

## Results

**Fig 1:**
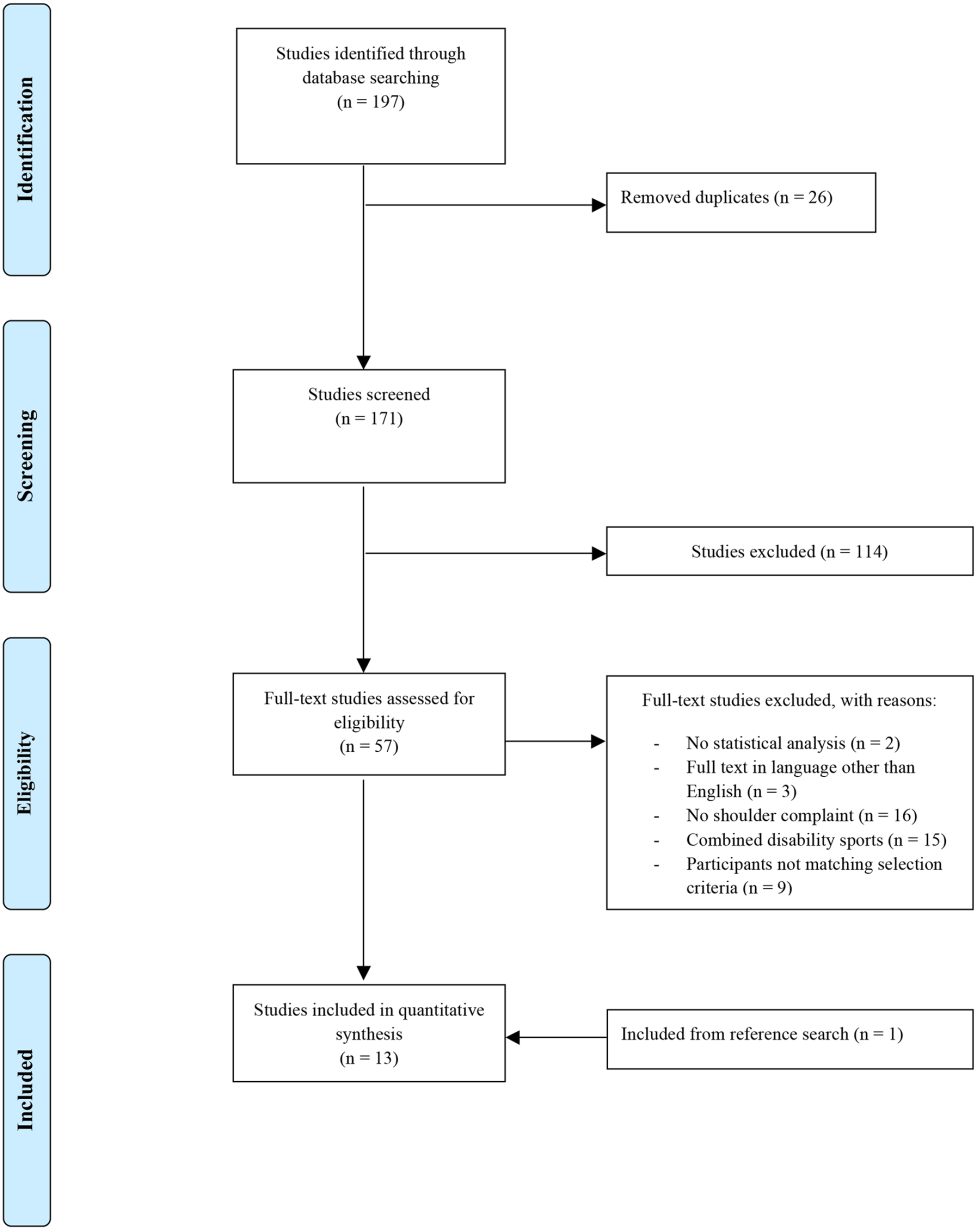
Flow chart describing the selection process of the included studies.

The combined search yielded 197 results, after removing duplicates 171 articles remained. The initial screening process excluded 114 articles, 57 full text articles were screened and 45 articles were excluded, 1 article was included via reference list scan (Fig 1). The final thirteen papers included in the review included seven epidemiological studies on shoulder pain [14,15,36–40], one on shoulder complaints [41], one on the relationship between shoulder strength to shoulder complaints [42], two epidemiological studies on sporting injuries in general [43,44], one risk analysis [45], and one study describing the development of a new index [46]. Eleven of the included articles were cross-sectional descriptive studies [14,15,36–42,45,46], the other one consisted of a prospective cohort study[43]. Six of the studies were published in the past decade [37,39–41,43,45].

All of the papers reported the prevalence of the studied population that was experiencing a shoulder complaint except for one [39] (Table 1). One paper described the incidence of complaints [43]. All papers but one [47] used self report in the form of survey, questionnaire or interview to collect data on shoulder complaints, five of these studies went on to perform an additional clinical evaluation of the shoulder complex to assess for pathology [15,36,41,42,45]. Three studies [37–39] solely used a reliable and validated measurement tool, the Wheelchair Users’ Shoulder Pain Index [48] (WUSPI).

**Table 1:** Summary of studies describing the subjects and athletic activities, where the samples were drawn from, the study designs, measurement tools, types of complaints, current prevalence, proposed mechanisms and potential preventative measures.

Author (year)	Sport	Sample size	M/F	Age^a^	Years of athletic activity^a^	Sample drawn from	Study design	Questionnaire / interview	Objective measure(s)	Clinical evaluation	Type of shoulder complaint (%)^b^	Proposed mechanism	Preventative measure
Akbar et al. [45]	Wheelchair basketball	103	86/17	49	X	SCI patients	Cross sectional	Yes	Radiographic analysis	Yes	RCT (76%)	Overuse	X
Burnham et al. [36]	Wheelchair athlete*	19	19/0	29	X	WA volunteers	Cross sectional	X	Isokinetic shoulder strength	Yes	RCI(26%)	Muscular imbalance	Strength training
Chung et al. [43]	Wheelchair fencing	14	7/7	29	10	HKWFF national squad	Prospective cohort	Yes	X	X	Shoulder strain (16%)	Upper limb over-compensation	X
Curtis & Black [38]	Wheelchair basketball	26	0/46	33	X	National women’s WB tournament participants	Cross sectional	Yes	WUSPI	X	Pain (52%)	Overuse	Flexibility and strength training
Curtis et al. [46]	Wheelchair athlete*	64	62/2	43	X	Paralyzed WA veterans	Cross sectional	Yes	Modified SPADI	X	Pain (73%)	X	X
Finley& Rogers [15]	Wheelchair athlete*	26	23/3	42	X	Various sources	Cross sectional	Yes	X	Yes	Pain (23%)	X	X
Fullerton et al. [14]	Wheelchair athlete*	172	X	X	10	Various sources	Cross sectional	Yes	X	X	Pain (39%)	X	Athletic activity
Jeon et al. [41]	Wheelchair tennis	33	26/7	36	8	International Wheelchair Tennis Open	Cross sectional	Yes	Radiographic analysis	Yes	Pain (70%); AC pathology (67%); RCT (24%)^c^&(18%)^d^; biceps tendon pathology (21%)^c^&(18%)^d^; sub-acromial/deltoid effusion (33%)^c^&(18%)^d^	Scapular dyskinesis; Overuse	Early detection and classification guidelines
Miyahara et al. [42]	Quadriplegic rugby	8	X	27	X	New Zealand Wheel Blacks Squad	Cross sectional	Yes	Isokinetic shoulder strength	Yes	Pain (100%); Shoulder impingement syndrome (50%)	Denervation of shoulder adductors	Shoulder adductor strengthening
Taylor & Williams [44]	Wheelchair racing	53	41/12	25–39^e^	3	BWRA members	Cross sectional	Yes	X	X	Shoulder (and upper arm) injury (25%)	Overuse	X
Tsunoda et al. [39]	Wheelchair basketball	40	19/21	29	10	Japanese WB National Team	Cross sectional	Yes	WUSPI; PC-WUSPI	X	Pain (X)	X	Daily care e.g. stretching
Ustunkaya et al. [40]	Wheelchair basketball	25	25/0	29	X	Professional Sport Clubs	Cross sectional	Yes	WUSPI; PC-WUSPU; functional tests; SWLS	X	Pain (44%)	X	X
Yildirim et al. [37]	Wheelchair basketball	60	X	25	6	WB volunteers	Cross sectional	Yes	WUSPI; PC-WUSPI	X	Pain (21%)^f^& (23%)^g^	Poor trunk control	Trunk stabilization

* = undefined sport;

^a^ = mean in years;

^b^ = % of population affected;

^c^ = dominant shoulder;

^d^ = non-dominant shoulder;

^e^ = 59% aged between 25 and 39 years;

^f^ = WA with high trunk control;

^g^ = WA with low trunk control;

AC = Acromio-clavicular; BWRA = British Wheelchair Racing Association; CE = Clinical Evaluation; HKWFF = Hong Kong Wheelchair Foil Fencing; NS = Non-Specified; PC-WUSPI = Performance Corrected Wheelchair Users’ Shoulder Pain Index; RCI = Rotator Cuff Impingement; RCT = Rotator Cuff Tear; SCI = Spinal Cord Injury; SPADI = Shoulder Pain and Disability Index; SWLS = Satisfaction with Life Scale; WA = Wheelchair Athletes; WUSPI = Wheelchair Users’ Shoulder Pain Index; X = not presented in reviewed paper.

### Quality of the evidence

The results of this review should be viewed with some discretion with respect to the level of evidence (Table 2). Seven of the papers adequately described participant characteristics [15,38–41,43,44], one paper fully described inclusion / exclusion criterion [45]. All papers had appropriate study designs based upon their research questions and measured key dependent variables and adequately provided details. There was a lack of reliable and valid measurement tools used in the selected papers, three papers satisfied the requirements based on the quality assessment checklist (S1 Table) [37–39]. Less than half of the papers generalised their findings to the other cohort populations [15,36,39,43,46]. Study limitations were discussed to varying extents, six authors described them inadequately or not at all [36,40,42,44,46,49]. From a maximum available score of 8 and a minimum available score of 0 the highest and lowest scores for the manuscripts included in this review were 7 [39] and 3 [14,42,44], respectively, with a total mean of 4.5 across all manuscripts.

**Table 2:** Quality assessment adapted from Webster et al. [23].

	1. Participant characteristics	2. Were inclusion / exclusion criteria stated?	3. Was the design appropriate to the research question?	4. Were key dependent variables measured?	5. Psychometric properties (reliability)	6. Psychometric properties (validity)	7. Was the external validity of the results discussed?	8. Were the limitations of the studies described?	Total Score
Fullerton et al.[14]	--	--	++	++	--	--	--	++	**3**
Miyahara et al. [42]	+-	--	++	++	+-	--	--	--	**3**
Taylor & Williams [44]	++	--	++	++	--	--	--	--	**3**
Burnham et al. [36]	+-	--	++	++	--	--	++	--	**3.5**
Curtis et al. [46]	+-	+-	++	++	--	--	++	--	**4**
Akbar et al. [45]	+-	++	++	++	--	--	--	++	**4.5**
Jeon et al. [41]	++	+-	++	++	--	--	--	++	**4.5**
Ustunkaya et al. [40]	++	+-	++	++	+-	+-	--	--	**4.5**
Chung et al. [43]	++	--	++	++	--	--	++	++	**5**
Yildirim et al. [37]	+-	--	++	++	++	++	--	+-	**5**
Finley & Rogers [15]	++	--	++	++	+-	--	++	++	**5.5**
Curtis & Black [38]	++	--	++	++	++	++	--	++	**6**
Tsunoda et al. [39]	++	--	++	++	++	++	++	++	**7**

++ = Yes/Adequately described; +- = Partial / Limited description; -- = Inadequately described /No/Not Stated

### Type of complaints

Shoulder pain was a common complaint in the wheelchair athletes studied in this review, all but one paper reported pain [43]. Complaints other than pain included rotator cuff impingement [36], rotator cuff tear [41,45], acromio-clavicular pathology [41], bicep tendon pathology [41], subacromial and subdeltoid effusion [41], non specific shoulder (and upper arm) injury [44] and shoulder strain [43]. Shoulder strain was not defined by Chung et al. [43]; it was diagnosed by a registered physiotherapist and an orthopedic surgeon and is assumed to mean a soft tissue strain of any of the musculature of the scapula-humero-thoracic system. The same authors also found tendon/ligament ruptures and sprains as upper-extremity complaints but did not specify the exact region. It is worthwhile to note that in the study by Burnham et al. [36] that even though pain was not directly measured, for a positive rotator cuff impingement assessment the athlete must have reported pain and at least two other clinical symptoms.

### Prevalence and incidence

The experience of current shoulder complaints varied greatly in percentage of the populations studied. Current shoulder complaints in wheelchair athletes ranged from 21% of 29 wheelchair basketball players with good trunk control [37] to 76% of 103 overhead sports wheelchair athletes [45]. Chung et al. [43] reported the incidence of shoulder and upper arm complaints to be 1.1 /1000 hours of participation in wheelchair foil fencing.

Pain since the start of wheelchair use was reported to range from 52% in wheelchair basketball players with good trunk control [37] to 72% in international level female wheelchair basketball players [38]. Presumably Finley and Rogers [15] found a lower percentage (33%) of athletes in their sample that experienced pain since the onset of wheelchair use, compared to Yildirim and colleagues [37]. This percentage was reported to be from the onset of athlete disability, this also happened to be the same time at which the athletes started using a wheelchair (20 years prior). Therefore it is assumed in the study of Finley and Rogers [15] that 33% of the wheelchair athletes in their study experienced pain since the onset of wheelchair use.

### Factors and underlying mechanisms

The cause of shoulder problems in wheelchair athletes is likely multifactorial and is difficult to identify. The selected papers in this review found that overuse [36,44], weakness in shoulder adduction, internal and external rotation [36] and decrease of trunk control [37] to be factors potentially involved in shoulder complaints in wheelchair athletes. Curtis and Black [38] discussed the possibility that poor driving posture in the wheelchair, with respect to arm elevation and torso positions during propulsion, may play a role in shoulder dysfunction. Jeon and colleagues [41] discussed the possibility of poor scapular kinematics, muscular imbalance and overuse as potential factors in the development of shoulder complaints. Some authors concluded that there is a lack of knowledge of the cause of complaints and that further research should be performed to better understand mechanism and factors involved [15,44,50].

### Preventative measures

Most of the papers in this review reported the prevalence of shoulder problems in wheelchair athletes but no paper directly looked at preventative measures. Nevertheless there were some recommendations to be drawn from their studies. According to Fullerton et al. [14] athletic activity protects the wheelchair athlete from shoulder complaints and exercise should be promoted in order to decrease shoulder pain. Other authors concluded that shoulder complaints were not influenced by athletic activities in wheelchair athletes [15,40,44]. Jeon and colleagues [41] suggested the development of guidelines for early detection, classification and treatment of shoulder injuries in wheelchair athletes in order to provide athletes with more pain free years. Two authors specifically mentioned preventative measures [38,39], but the recommendations were indistinct, i.e. to engage in stretching, general flexibility and strength training to prevent muscular imbalance and impingement. Strength training of the shoulder adductors, internal and external rotators was recommended as a rehabilitative treatment [36], only to be performed once the athlete has already been diagnosed with a problem. The rehabilitation training exercises, according to Burnham et al. [36], should be performed with the arms below the height of the shoulders to prevent the risk of impingement. The authors only briefly mentioned that strength training of the shoulder complex adductors may be a potential prophylactic measure.

## Discussion

In this literature review an attempt is made to grasp the problem of shoulder complaints in wheelchair athletes in terms of understanding and describing the extent of the problem, examining the underlying factors and possible mechanisms involved; and initiating an introduction to potential preventative measures to decrease the risk of developing problems in the first case.

Overall there is a lack of current research in wheelchair and disabled athletes. It was found in this review, that in general there were a relatively low number of subjects studied, a limited number of studies, a high number of cross-sectional studies and the prevalence of complaints was reported to be relatively high and was quite variable. Pain was the most common complaint. Preventative measures were indistinct and there may be a role for strength training implementation but further research is necessary.

### Prevalence and incidence

Pain was found as a common shoulder complaint in this review, six of the twelve manuscripts studied had research questions specifically looking to assess or compare shoulder pain (between two sub populations) [14,37–40,46]. It is possible that pain is such a frequently cited shoulder complaint because that is largely what researchers have been exclusively seeking. An approach that is guided by attempting to understand shoulder complaints via diagnosis may be superior in its ability to direct researchers to the underlying structural mechanism in these populations. Approaches that purely cite pain without delving into the underlying mechanism or cause may not aid in attempts to decrease the occurrence of pain.

Two authors took the diagnostic approach [15,41], they cited pain as a type of shoulder complaint but did not exclusively search for shoulder pain, instead they studied shoulder pathology. Finley and Rodgers [15] identified manual wheelchair users that experienced pain, these users then underwent a clinical evaluation for diagnosis. When reporting their findings (50% of the participants presented with bicipital tendonitis and 44% with impingement syndrome) they did not distinguish between athletic and non-athletic manual wheelchair users. Due to this fact, it is not possible to describe any pathology for wheelchair athletes. Future studies should consider this diagnostic approach but ensure discrimination between the two subpopulations when diagnosing and reporting injuries in order to better understand the problem. In the study of Jeon and colleagues [41] each participant underwent an upper-extremity clinical evaluation then a sonographic evaluation of the long head of the biceps tendon, the acromio-clavicular joint and rotator cuff tendons. The results of this study found that acromio-clavicular pathology (64%) was the most common finding in the dominant shoulder of wheelchair tennis athletes. Full-thickness rotator cuff tears were present in dominant (24%) and non-dominant shoulders (18%). Biceps tendon pathology was present in dominant (21%) and non-dominant shoulders (18%). Subacromial and subdeltoid effusion was present in dominant (33%) and non-dominant shoulders (18%). These results were found in symptomatic as well as asymptomatic athletes.

A large range in prevalence of shoulder complaints is seen in the athletic wheelchair population, varying from 16% [43] to 76% [45]. This large range of complaints in this population may be due to a number of factors including diverse study designs, slight differences in the precise information being reported, and utilization of various measurement tools. With cross-sectional studies there may be some bias based on the sampling time, there is a possibility that results would vary if the data were collected in another time frame. With respect to the information being reported by researchers, in cross-sectional studies all authors except one [39] reported current complaints, some additionally reported complaints since wheelchair use [37,38,40] or prior to wheelchair use [37,38,40]. On the other hand, in cohort studies the complaint prevalence is being reported for the entire data collection period. These factors may be additional reasons for the discrepancy in reported prevalence data in the literature.

Furthermore, the use of different measurement tools in the studies may also be a confounding factor that influences the large range of complaints in wheelchair athletes. As previously mentioned, three authors solely used reliable and valid measurement tools to collect their data [37–39]. The extensive use of self-report data in the literature may prove to be a weakness as there are numerous limitations of self-report data. These limitations may include participant selective memory, introspective ability, honesty and response bias [51].

Incidence data is often lacking in studies of this nature. The limit in incidence data available for this review is due to the study design chosen by the researchers of the included literature. Cross-sectional studies are unable to measure incidence rates as opposed to cohort studies. The single (prospective) cohort study [43] that included incidence rates reported an incidence of shoulder and upper arm injury at 1.1 per 1000 hours of participation. The authors did not differentiate between shoulder and other upper arm problems so this must be taken into account when reviewing shoulder complaints in this population. The incidence of shoulder injury alone is likely considerably lower because the highest reported problem in this population was elbow strain (33%). It would be beneficial for future research to include cohort studies to be able to incorporate incidence data so we could get a better understanding of shoulder complaints in wheelchair athletes.

### Factors and underlying mechanism

The cause of shoulder complaints in wheelchair athletes is likely to be multi-factorial, although given the study design limitations, with the abundance of cross-sectional studies found in this review, no causal factual relations or associations can be drawn. The literature on the topic is difficult to navigate at best; there is no general consensus to the problem at hand. This lack of consensus could be due to the lack of structure within the field with respect to a conceptual construct. Some authors advocate that shoulder complaints are alleviated or at least not aggravated by athletic activity [14,15,40,44], and promote wheelchair athletes to stay active. Conversely, others suggest that complaints may arise from overuse and/or muscular imbalance [36–38,41,45] among other factors. There may be a middle ground to be sought in which a balance is found between undertaking enough physical activity to enjoy the associated health benefits, and not overloading the shoulder complex to the point of muscular overuse or imbalance.

Overuse and muscular imbalance of the shoulder complex seem to be viable factors affecting wheelchair athletes. From biomechanical modeling studies the repetitive nature of handrim propulsion and the high biomechanical loads are thought to be causes that lead to overuse type injuries [8,9,11]. High glenohumeral joint contact forces during handrim propulsion have been measured during wheelchair propulsion. The high peak muscle force in supraspinatus, infraspinatus and biceps during the push phase, the start and end of recovery phase may lead to fatigue of the rotator cuff. Supraspinatus and infraspinatus both make up components of the rotator cuff. The excessive fatigue of these muscles has been hypothesized to lead this musculature to decrease their function of counteracting extreme superior humeral head translation [8]. This excessive superior translation of the humeral head occurs in conjunction with a decrease in subacromial space which has been associated with subacromial pain syndrome [52].

Two papers compared wheelchair athletes with high and low trunk control [37,43]. They both found that wheelchair athletes with low trunk control experienced more shoulder complaints than their high trunk control counterparts. The reasons the authors provide for this are varied, including a lack of congruency in the kinetic chain [43], overloading of the shoulder complex, and altered sitting posture [37]. The link between these two papers with respect to the underlying mechanism is that these three factors all eventuate to overuse of the shoulder complex. If trunk control is low, and the kinetic chain is limited from the ground up, the upper body has to compensate which could increase the risk of shoulder complaints [43]. Manual wheelchair users with low trunk control tend to sit in an altered sitting position compared with users with good trunk control [53]. It has been shown that altered posture disrupts scapula-humero-thoracic rhythm [54]. Thus wheelchair athletes with low trunk control could experience an increase in complaints in comparison to wheelchair athletes with good trunk control. On the other hand, Janssen-Potten et al. [55] has shown that an altered sitting posture may be beneficial. Their study showed that a seat that had forward inclination may decrease fatigue for manual wheelchair users that had to sit for extended periods of time. Balance was not affected by the variation in seat angle.

In many of these studies the factors and underlying mechanisms were not directly studied; the authors made educated assumptions based on the current literature and findings. This highlights a current lack of knowledge in the mechanism of shoulder complaints in wheelchair athletes that must be addressed to confidently deal with the problem. Much of the literature on shoulder complaints has been on pain prevalence in certain populations. What is required now is to examine the cause of these complaints in the wheelchair athletic population to be able to develop and introduce effective preventative measures to be applied in practice.

With respect to factors involved in shoulder complaints in wheelchair athletes, a distinction must be made between ambulant and non-ambulant manual wheelchair users. Manual wheelchair users that use the wheelchair for propulsion in addition to ADL activities experience extra loads in the shoulder complex. ADL tasks like weight relief lifts and negotiating curbs have been shown to create significantly higher net moments in the shoulder when compared with wheelchair propulsion [9]. This increased load could lead to more shoulder complaints in non-ambulant manual wheelchair users than ambulant manual wheelchair users [7,13]. Although a meta-analysis was not performed, results indicate that due to the amplified loading and consequent inherent increased risk for shoulder complaints experienced by non-ambulant users, future research should discriminate between ambulant and non-ambulant manual wheelchair users.

It is clear that there are many potential underlying factors that are responsible for influencing shoulder complaints in wheelchair athletes. Through studying the proposed associations between characteristics of the task and the user (Table 3) [9,11,14,17–20,56–59] we might be able to get deeper insight into the potential causal mechanisms of shoulder complaints.

**Table 3:** Association between task and user factors and complaints.

Factors		Complaints
**User**	Years of disability ↑	↑
	Age ↑	↑
	Gender (Female)	↔
	BMI ↑	↑
	Training status ↑	↔
**Task**	Wheel propulsion ↑	↔
	ADLs ↑	↔
	Sports activities ↑	↔

↑ = increase in factor or complaint; ↔ = indistinct association to complaints

User characteristics have been previously described in this article and for the most part seem to play a prominent role in the expression of complaints in wheelchair athletes. Increases in disability, age and BMI have been associated with an increase in complaints [17–19,58,59]. Being of the female gender or an increase in training status have not been definitively described to have a positive or negative effect on shoulder complaints [14,20]. Task characteristics on the other hand have no clear association to complaints. ADLs, as previously mentioned, have been shown to increase shoulder load but haven’t been directly linked with shoulder complaints [9]. Wheelchair propulsion and sports activities such as ball handling and overhead activities (throwing, shooting etc.) are known to load the shoulder but it is not known if this is a cause of complaints in wheelchair athletes [11,14,15,56,57]. The mechanisms proposed above are not an exhaustive description and need to be confirmed with future research.

**Fig 2:**
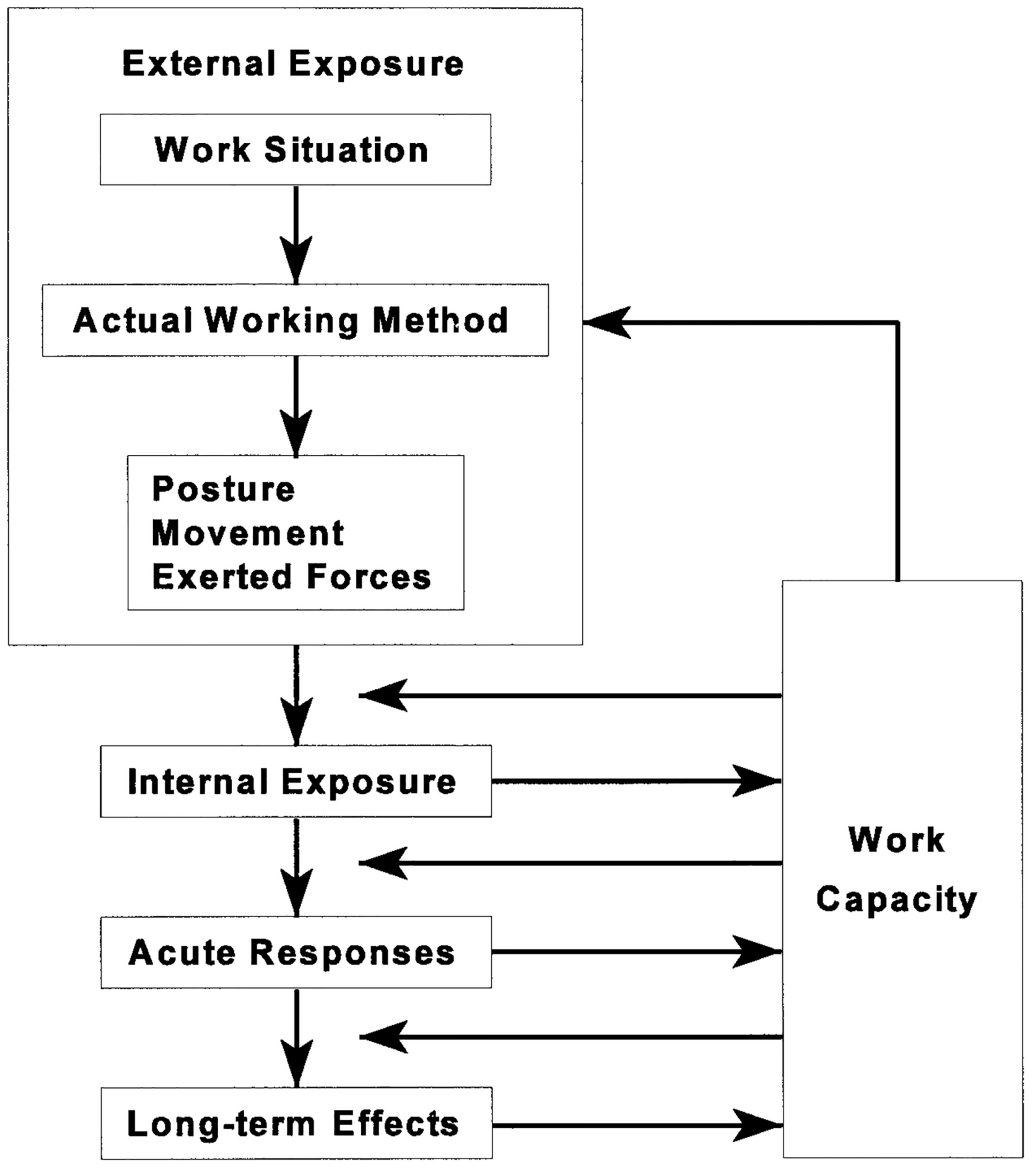
Conceptual model described by Hoozemans et al. [7], based upon the model of Westgaard and Winkel [33] and the model of Van Dijk [34].

Research in this area lacks organization, there might be potential in approaching the problem from a different angle to further deepen knowledge. What the included studies are missing and what research could benefit from in this area is a mechanistic breakdown of load in terms of intensity, frequency and duration of force during various athletic tasks. A conceptual framework in which external exposure is related to internal exposure, which eventuates to certain long-term outcomes was described by Hoozemans et al. [7] in occupational research, but based upon the work of Westgaard and Winkel [33] and Van Dijk [34] (Fig 2). In this model, a work situation (e.g. wheelchair athletics) can be performed by means of the actual working method (e.g. wheelchair pushing technique, ball handling etc.) which in turn leads to certain body postures, movements and exerted forces. The combination of work situation, actual working method; and posture, movement and exerted forces combined compromise the external exposure. External exposure precedes internal exposure [33], which may come in the form of increased load at the shoulder complex. The resultant effects are acute, short term physical and mental responses. These acute responses can lead to positive or negative long term effects (e.g. shoulder complaints, improved physical capacity). The athletes work capacity plays a major role in the model and has an inverse relation to a multitude of factors. Work capacity is reliant not only upon the athletes’ physical prowess and characteristics but also upon their mental attributes and capacities.

The work of Hoozemans et al. [7], performed in occupational research, may be able to shed some light on how to approach the current problem of shoulder complaints in wheelchair athletes. Hoozemans and his colleagues examined the characteristics of load from four different perspectives; namely epidemiology, psychophysics, physiology and biomechanics. With this approach they were able to fit these perspectives into his model and describe the issue from various standpoints. The epidemiological perspective examined the relationship between external exposure and long-term effects; psychophysical and physiological perspectives were concerned with the effect of external exposure on acute responses; and the biomechanical perspective examined the relationship between external and internal exposure.

The endeavor of describing and understanding the problem of shoulder complaints in wheelchair athletes should perhaps also be examined through the combination of these various perspectives, within this conceptual model in order to organize the process and to deepen our comprehension of the current concern within the larger context.

Biomechanical models are basic representations of the human body that can be used to predict the load on the human body, its joints and structures. Currently load is a ‘black box’ concept that is poorly understood within the context of shoulder complaints in wheelchair athletes. There would be benefit in incorporating some practices of Hoozemans et al. [7] by dissecting load into the previously mentioned factors of intensity (amplitude and direction), frequency, and duration. By tackling each of these factors separately via means of biomechanical modelling studies perhaps as researchers we can find a way to open this black box and unravel the complexity that is load.

### Preventative measures

General shoulder strengthening regimens have been recommended to wheelchair athletes for decreasing the risk of injury to the shoulder complex for decades [3]. Curtis et al [60] developed an effective rehabilitation protocol involving three exercises for strengthening the posterior shoulder and two stretches for the anterior shoulder based upon the theory that the repetitive nature of the wheelchair stroke pattern causes imbalance and subacromial pain. The participants of this study were not wheelchair athletes but community dwelling long-term (14±9 years) manual wheelchair users. As the research is sparse and inconclusive on injury mechanism within this subpopulation there have therefore been no preventative protocols developed and tested.

The study of Akbar and colleagues [45] brings an important point to light and concludes that the current dilemma is now to find a way to increase physical activity in manual wheelchair users without increasing the risk of upper extremity overuse. A balance must be found between the two in order to optimize QoL. Handcycling may provide some insight into how this dilemma may be solved. What the research tells us is that the nature of manual wheelchair propulsion puts a greater deal of strain on the shoulder complex and is less efficient when compared to handcycling [61]. The manual wheelchair produces large spikes in glenohumeral contact forces; this is especially prevalent during the initial push phase. The handcycle on the other hand allows the user to evenly distribute glenohumeral contact force over the entire duration of the propulsion cycle [8]. Handcycling emphasizes a circular cycle that emphasizes flexors and extensors as opposed to the push-recovery cycle of the manual wheelchair [62]. Manual wheelchair users may be at risk for overuse injury due to the imbalanced nature of the movement. Muscular force in the rotator cuff, especially in the supraspinatus, are greatly reduced with a handcycle [8]. This raises the possibility of including some phases of handcycling into the yearly training cycle for some wheelchair athletes in order to decrease overall shoulder strain. Obviously for athletes competing in manual wheelchairs, training specificity becomes a major consideration. However, perhaps in the general preparation phases, where there are no impending competitions, there may be a place for handcycle implementation in order to decrease risk of shoulder complaints.

In an attempt to draw some conclusions about complaint prevention measures in wheelchair athletes, it was deemed necessary to delve into prevention literature for other disabled sports. There are also large knowledge gaps in this area. In disabled swimming overuse complaints have been reported at 80% and the shoulder is the most affected body region [63]. This seems to be due to the nature of the sport, not the disability. Proposed prevention strategies include monitoring and adjusting training load to decrease risk of overuse injury. In winter Paralympic sports including disabled alpine skiing, Nordic skiing and sledge hockey the injury profiles have been reported to be very similar to those of able-bodied athletes in similar disciplines. Most of these injuries were of the acute nature. Across the three winter sports a minimum of 33% of the included injuries involved the upper limb; unfortunately the exact area of the upper limb was not specified [64].

From the current research it seems that to decrease risk of injury or complaint to the shoulder complex one must manage load and decrease shoulder strain. There must be some balance found between enjoying the positive benefits of physical activity without overloading the shoulder complex. This may prove to be a challenge for the competitive wheelchair athlete, especially a non-ambulant wheelchair athlete. An avenue that may be effective and warrants deeper investigation for the application to wheelchair athletes is a well-structured strength training program that could help to counterbalance the repetitive nature of the wheelchair stroke pattern.

Future research should focus on the systematic longitudinal surveying of individual and groups of wheelchair athletes by trained physicians in attempt to deduce some kind of pattern of the consequences of impairments secondary to wheelchair use within these populations. It is of the utmost importance to gain an understanding of injury mechanism within this population in the long term to be able to develop preliminary prevention strategies. Modelling studies should be performed in order to biomechanically and systematically investigate, evaluate and dissect load during different athletic tasks and ascertain the effect of external exposure upon internal exposure.

### Limitations

Firstly, as a result of the lack of high quality literature available to be included in this review it was necessary to delve into literature in non-athletic manual wheelchair users, able-bodied populations, industrial ergonomics and occupational research to obtain a firmer grasp on potential underlying factors, possible mechanisms, prevention strategies and research frameworks. This may have limited the generalisability to wheelchair athletes but until there is a solid base of existing current literature we have limited options in order to understand the problem. Secondly, since most of the papers in this literature review were cross-sectional studies it is important to note that it is impossible to make any causal inferences due to the nature of the study designs.

## Conclusion and practical implications

It can be seen from the results of this review that shoulder complaints in wheelchair athletes is a common problem that needs to be addressed in the literature and in practice. The studies included in this review found there is a high prevalence of shoulder complaints, especially of pain, in wheelchair athletes. The incidence, mechanism and underlying factors are not clearly understood and therefore although it is impossible to introduce effective preventative measures, it is plausible that a balance must be found between enjoying the benefit of physical activity and minimizing overload of the shoulder complex. Future research should make an attempt to bridge the gap between what is known about able-bodied persons, non-athletic manual wheelchair users and other areas of occupational ergonomics to athletic manual wheelchair users. The importance of sharing knowledge from various perspectives should be stressed. Research should be directed towards biomechanical modeling to develop knowledge of load and its effects. Longitudinal study designs should be performed to gain an understanding of the incidence and underlying factors involved in shoulder complaints in wheelchair athletes so that effective preventative measures can be developed. These studies should differentiate between ambulatory and non-ambulatory manual wheelchair users due to the effects of ADL activities on the user. It is the culmination of perspectives that will allow full realization of the extent of complaints in wheelchair athletes.

## Supporting information

S1 Table Quality assessment criteria checklist. Assessment of quality was performed independently with an adapted version of a checklist (S1 Table) developed by Webster et al.[23].
(DOCX)

S2 Table PRISMA checklist. The filled in PRISMA checklist for ‘Shoulder complaints in wheelchair athletes: A systematic review’.
(DOC)
