# Supplementary material for: Shoulder complaints in wheelchair athletes: A systematic review
Source: PLoS One. 2017 Nov 21;12(11):e0188410. doi: 10.1371/journal.pone.0188410 (PMC5697842; doi:10.1371/journal.pone.0188410)
Supplement: S1 Table — Assessment of quality was performed independently with an adapted version of a checklist (S1 Table) developed by Webster et al.[23]. (DOCX) [file pone.0188410.s001.docx]

**Appendix 1**

Quality assessment criteria checklist adapted from Webster et al. [24].

| **Questions** | **Response / decision rule criteria** |
| --- | --- |
| 1. Participant characteristics – are participant demographics adequately described? (include: sport, level of competition, number, age, gender) | Adequate – all details provided  Partial – all details except level of competition or gender  Inadequate – missing details |
| 2. Were inclusion/exclusion criteria stated? | Stated - clear list of both given  Limited - one or two points only  Not stated - not provided |
| 3. Was the design appropriate to the research question? | Yes - well matched to question  No |
| 4. Were key-dependent variables measured? | Adequate - all details provided  Partial - only some variables measured  Inadequate - missing details |
| 5. Psychometric properties - was the reliability of measurement tools reported and adequate? | Adequate - all details provided  Partial - only some aspects of reliability reported  Inadequate - missing details |
| 6. Psychometric properties —was the validity of measurement tools reported and adequate? | Adequate - all details provided  Partial - only some aspects of validity reported  Inadequate - missing details |
| 7. Was the external validity of the results discussed? | Yes - generalisability of findings discussed  No |
| 8. Were the limitations of the studies described? | Adequate - all limitations discussed  Partial - limited description  Inadequate - not described |
